# Nc2Eye: A Curated ncRNAomics Knowledgebase for Bridging Basic and Clinical Research in Eye Diseases

**DOI:** 10.3389/fcell.2020.00075

**Published:** 2020-02-14

**Authors:** Yan Zhang, Zhengbo Xue, Fangjie Guo, Fulong Yu, Liangde Xu, Hao Chen

**Affiliations:** School of Biomedical Engineering, School of Ophthalmology and Optometry and Eye Hospital, Wenzhou Medical University, Wenzhou, China

**Keywords:** eye diseases, non-coding RNAs, knowledgebase, epigenomics, website

## Abstract

Eye diseases (EDs) represent a group of disorders affecting the visual system, most of which can lead to visual impairment and blindness. Accumulating evidence reveals that non-coding RNAs (ncRNAs) are closely associated with a wide variety of EDs. However, abundant associations between ncRNAs and EDs are scattered across the published literature, obstructing a global view of ncRNA-ED associations. A public resource of high-quality manually curated ncRNAomics knowledge associated with EDs remains unavailable. To address this gap, we thus developed Nc2Eye (http://nc2eye.bio-data.cn/), which is the first knowledgebase dedicated to providing a comprehensive ncRNAomics resource for bridging basic and clinical research in EDs. Through a comprehensive review of more than 2400 published papers, Nc2Eye catalogs 7088 manually curated ncRNA-ED associations involving 4363 ncRNAs across eight species. We also provide detailed descriptions and annotation information for each ncRNA-disease association such as ncRNA categories, experimental methods, expression pattern and related clinical drugs. To further expand the pathogenic ncRNAs, we also collected more than 90 high-throughput EDs-related transcriptome datasets. Furthermore, a user-friendly interface was constructed for convenient and flexible data browsing, querying, and retrieving. We believe that Nc2Eye is a timely and valuable knowledgebase for significantly improving and useful for discovery of new diagnostic and therapeutic biomarkers.

## Introduction

Eye diseases (EDs) represent a group of disorders affecting the visual system. It was estimated that 217 million people (3.1% of the world population) suffered from moderate to severe visual disturbance in the world in 2015, while 36 million were blind (Bourne et al., 2017). There are more than 150 kinds of EDs recorded in medical subject headings (MeSH) (Bhattacharya et al., 2011), most of which can lead to visual impairment and blindness, such as cataract, age-related macular degeneration (AMD), diabetic retinopathy (DR), retinoblastoma (RB), and degenerative myopia, etc. The pathogenesis of EDs at the molecular level remains poorly understood.

Non-coding RNAs (ncRNAs), such as microRNA (miRNA), long non-codingRNA (lncRNA) and circlar RNA (circRNA), are a large category of functional RNA molecules involved in regulating many biological processes (Esteller, 2011). Numerous studies demonstrated that ncRNAs play important roles in various EDs (Li et al., 2016; Rassi et al., 2017; Zhang et al., 2017). For example, the lncRNA *PVT1* is up-regulated in RB tissues and silencing *PVT1* can suppress the tumor growth (Wu et al., 2019). Overexpression of circRNA *cPWWP2A* was reported to alleviate retinal vascular dysfunction in DR by inhibiting *miR-579* activity (Liu et al., 2019). *miR-27a* is up-regulated in uveal melanoma cell lines (Venza et al., 2014) and Sun et al. (2009) found that genistein inhibited uveal melanoma cell proliferation in part through decreasing *miR-27a* expression. These studies suggested that ncRNAs not only contribute to the understanding of the molecular mechanisms of EDs, but also have important implications for the development of new therapeutic targets.

With the progression of disease-related ncRNA study, some disease-associated ncRNA resources were constructed, such as LncRNADisease (Chen et al., 2013), miR2Disease (Jiang et al., 2009), and MNDR (Cui et al., 2018). These databases contained a wide variety of human diseases and there were only a few ncRNAs involved in EDs. Subsequently, several ophthalmology-related databases were built recently. For example, KmeyeDB (Kawamura et al., 2010) and RetinoGenetics (Ran et al., 2014) provided gene mutations in EDs. miRNeye (Karali et al., 2010) focused on the differentially expressed miRNAs in ocular tissues. iSyTE (Kakrana et al., 2018) integrated all publicly available lens gene expression data. However, there is still no an ED-specialized database that provides the comprehensive resource on diverse types of ncRNAs across various EDs.

To fill the gap, we constructed Nc2Eye, a manually curated database, to provide experimentally validated ncRNA-ED associations. The current version of Nc2Eye contains 7088 associations between 104 EDs and 4363 ncRNAs in eight species, through a comprehensive review of more than 2400 published papers. To investigate the underlying molecular mechanism of EDs, Nc2Eye gathered more than 90 high-throughput transcriptome datasets in EDs, and normal controls. In addition, Nc2Eye provided a user-friendly interface for convenient and flexible data browsing, querying, retrieving and submitting. Nc2Eye is a timely and valuable resource to significantly improve our understanding of ncRNA dysfunction in EDs.

## Data Collection and Database Content

### Experimentally Supported ncRNA-ED Associations

To ensure the accuracy and reliability in the data collection process, we manually curated all Nc2Eye entries by the following steps. First, we searched the PubMed database (Sayers et al., 2019) with the MeSH term “EDs” and a list of keywords about ncRNAs, including “ncRNA,” “non-coding RNA,” “non-coding,” “lncRNA,” “long non-coding RNA,” “miRNA,” “microRNA,” “siRNA,” “shRNA,” “snoRNA,” “piRNA,” “circular RNA,” “circRNA,” “miR-,” “and “let-.” Considering that some papers have not been assigned MeSH term, we chose the eye disease names from MeSH term as keywords. Then, we searched with the keyword combination: each eye disease and ncRNA category name as complementary. After that, we selected published papers about the ED-related ncRNAs and extracted experimentally supported ncRNA-ED associations manually. All selected literature was reviewed by at least two researchers. In this step, we retrieved the ncRNA name, ncRNA category (e.g., miRNA, lncRNA, and circRNA), disease name, species, tissues/cell line, methods (e.g., microarray, qPCR, and Western blot), expression pattern (e.g., up-regulated, down-regulated), functional description and reference (PubMed ID, year of publication, title). In addition, we recorded whether the ncRNA was related to drugs according to the paper. Finally, we standardized the eye disease names refer to the MeSH term and searched the gene ID, Ensembl ID, and synonyms of each lncRNA from Ensembl (Zerbino et al., 2018) and NCBI Gene database (Brown et al., 2015).

After comprehensively reviewing more than 2400 published papers, a total of 7088 associations between 104 EDs and 4363 ncRNAs in eight species were manually collected. There were 132 of these ncRNAs were reported to be related with drugs in 14 EDs. Distribution of ncRNA-associated entries in each species and ncRNA category is listed in Table 1.

**TABLE 1:** Statistics for the ncRNA-ED entries in the Nc2Eye database.

**Species**	**miRNA**	**lncRNA**	**circRNA**	**siRNA**	**shRNA**	**piRNA**	**snoRNA**	**snRNA**	**Total**
Homo sapiens	3044	968	479	274	48	18	–	2	4833
*Mus musculus*	1016	165	2	137	41	–	4	1	1366
*Rattus norvegicus*	623	9	2	75	24	–	–	–	733
*Canis familiaris*	47	–	–	–	2	–	–	–	49
*Macaca mulatta*	17	5	–	14	3	–	–	–	39
*Bos taurus*	8	–	–	17	1	–	–	–	26
*Oryctolagus cuniculus*	1	–	–	21	–	–	–	–	22
*Danio rerio*	20	–	–	–	–	–	–	–	20
Total	4776	1147	483	538	119	18	4	3	7088

### High-Throughput Transcriptome Datasets in EDs

A great number of studies based on high-throughput experiments have emerged in recent years (Liu X. et al., 2018; Liu Y. et al., 2018; Xu et al., 2018). Mining and collecting high-throughput transcriptome datasets will help to investigate the underlying molecular mechanism of EDs. Nc2Eye gathered 91 microarray and next-generation sequencing datasets in EDs versus normal controls, most of which come from gene expression omnibus (GEO) database. Each entry contains “diseases name,” “species,” “sample type,” “case/control description,” “sample size,” “data type,” “accession ID,” “platform,” and “Pubmed ID.” Users can conveniently browse and select transcriptome datasets according to their research requirements and click the GSE ID to jump to the GEO website for further exploration. GEO has developed several tools for data visualization and analysis which enable researchers to easily analyze this data and do not require to download any file (Clough and Barrett, 2016).

Finally, all data in Nc2Eye were organized using MySQL (version 5.7.26). The web interface was built in PHP and the web services were deployed using Apache (version 2.4.6). The Nc2Eye database is freely available at http://nc2eye.bio-data.cn/.

## User Interface

**FIGURE 1:**
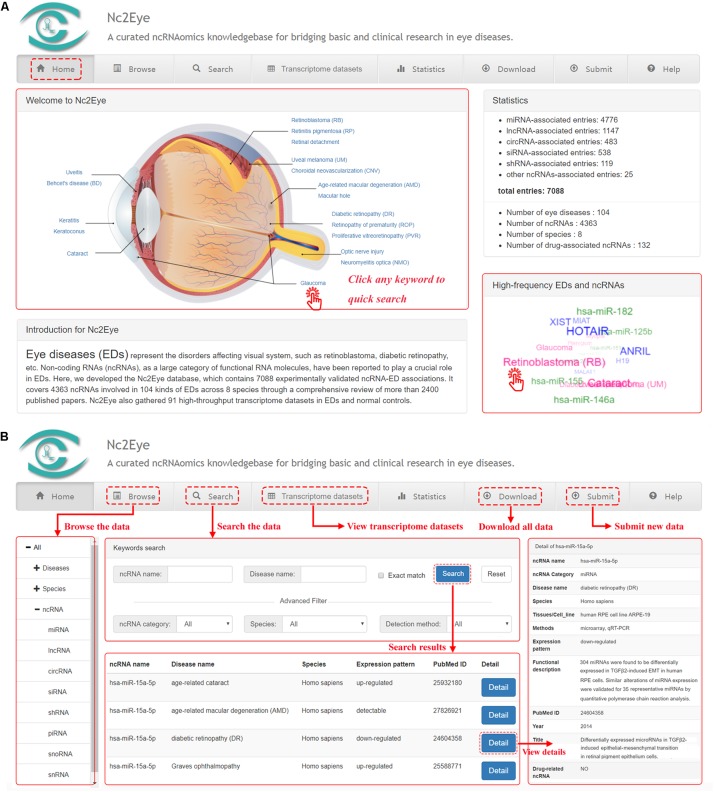
A schematic workflow of Nc2Eye. **(A)** The ‘Home’ page allow to quick research for ncRNA-ED associations. **(B)** The ‘Browse’ and ‘Search’ pages allow the users to browse and search ncRNA-ED associations. The ‘Transcriptome datasets’ page shows public high-throughput transcriptome datasets. Users can download all ncRNA-ED association data in the ‘Download’ page and submit new ncRNA-ED association in the ‘Submit’ page.

Nc2Eye constructed a user-friendly web interface for convenient and flexible data browsing, querying, retrieving and submitting (Figure 1). In the “Home” page, users can click keywords in the eye anatomy and “high-frequency EDs and ncRNAs’ to quickly research. In the “Browse” page, users can glance through Nc2Eye by clicking on a specific ED name, species or a class of ncRNA, and a list of corresponding entries will be displayed. Nc2Eye provides a “Search” page that enables users to search by ncRNA name, ED name or both. Defaulting to fuzzy search, and Nc2Eye also supports exact search. Moreover, users can use the “Advanced Filter” to filter the results by ncRNA category, species and detection method. In the “Transcriptome datasets” page, 91 expression profiles including EDs versus normal controls are listed and can be filtered by inputting keywords from any column. Nc2Eye also provides a submission interface that allows researchers to submit new ncRNA-ED association data which is not documented. At last, all ncRNA-ED association data in Nc2Eye can be downloaded in the “Download” page and a user tutorial of the website is available in the “Help” page.

## Discussion and Conclusion

Eye diseases are a common group of disorders in the visual system, causing visual impairment and blindness. Emerging evidence demonstrated that the dysregulation of ncRNAs played critical roles in various EDs. We developed Nc2Eye, a curated knowledgebase of experimentally validated ncRNAs associated with EDs, which provides a global landscape of ncRNAs in EDs.

**FIGURE 2:**
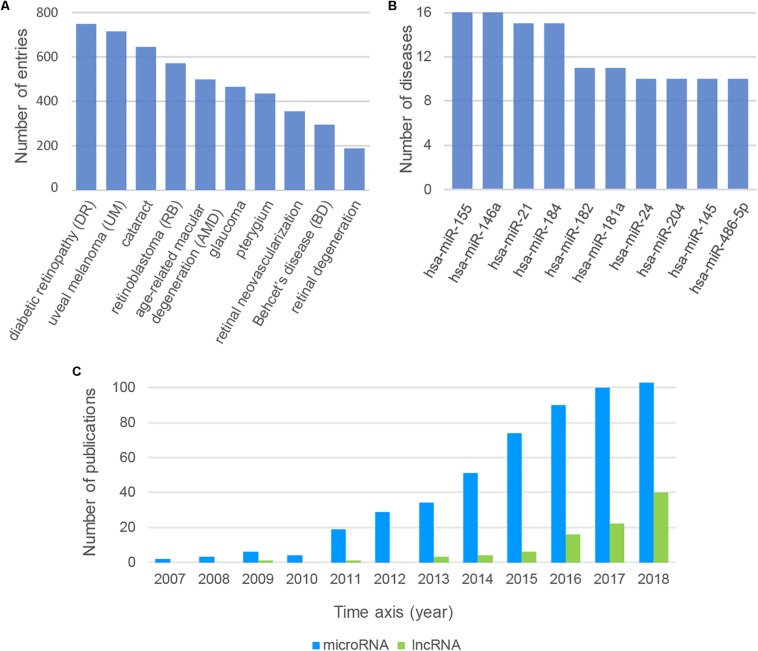
**(A)** The top10 diseases with the most ncRNA associations. **(B)** The top10 ncRNAs with the most disease relationships. **(C)** The number of ncRNA-ED publications each year.

We could find some important messages behind the large and complex data resources by analyzing the data from Nc2Eye. Top10 diseases with the most ncRNA associations were listed (Figure 2A). Diabetic retinopathy (DR) ranked first, which is associated with 583 ncRNAs, including 32 drug-related ncRNAs. This result suggested that we can develop more drugs to treat DR by targeting these ncRNAs. The top10 ncRNAs with the most disease relationships were shown in Figure 2B. The *hsa-miR-155* has the most connection with diseases and it is associated with 16 diseases, such as DR and RB, indicating the importance of this miRNA in ophthalmopathy. In addition, we tracked the number of ncRNA-ED publications each year (Figure 2C) and found the research about lncRNA is increasing year by year which implies lncRNAs will become a hot topic in ophthalmopathy.

## Conclusion

In conclusion, Nc2Eye, an eye disorders-specialized database, provides global insights into ncRNA functions in various EDs. We believe that Nc2Eye will be beneficial to researchers to dissect the underlying mechanism of ophthalmopathy. In the future, a confidence score system will be developed to estimate the reliability of a specific ncRNA-ED association according to experimental evidence. In addition, we plan to update the database every 3 months to extend newly ED-related relationships and make it more powerful.

## Data Availability Statement

Publicly available datasets were analyzed in this study. This data can be found here: http://nc2eye.bio-data.cn/download.html.

## Author Contributions

HC and LX conceived and designed the experiments. YZ, ZX, and FY collected and analyzed the data. FG designed the website. YZ and LX wrote and revised the manuscript. All authors read and approved the final manuscript.

## Conflict of Interest

The authors declare that the research was conducted in the absence of any commercial or financial relationships that could be construed as a potential conflict of interest.
